# Does SARS-CoV-2 infect platelets?

**DOI:** 10.3389/fimmu.2024.1392000

**Published:** 2024-04-23

**Authors:** Saravanan Subramaniam, Naila Mohiuddin, Asha Jose

**Affiliations:** ^1^ Department of Pharmacology and Toxicology, School of Pharmacy, Massachusetts College of Pharmacy and Health Sciences, Boston, MA, United States; ^2^ Renal Section, Department of Medicine, Boston Medical Center, Boston University School of Medicine, Boston, MA, United States

**Keywords:** platelets, COVID-19, inflammation, protease-activated receptors, coagulation

## Introduction

COVID-19 is characterized by dysregulated thrombo-inflammatory responses and coagulation, leading to an increased risk of mortality in affected patients (1–3). Platelets play a crucial role as rapid responders to the presence of pathogens, alerting nearby immune cells and contributing to intravascular coagulation (4). In cases of Acute Respiratory Distress Syndrome (ARDS), respiratory failure and thrombocytopenia account for 70% of COVID-19-related deaths (5). The remaining fatalities result from a sepsis-like condition triggered by a cytokine storm in response to viral infection and secondary infections (6). Various studies have demonstrated that COVID-19 patients have elevated levels of inflammatory cytokines, including IL-6, TNF-α, IL-1β, and IL-18 (7, 8).

Administration of steroids or anti-IL-6 monoclonal antibodies has been associated with increased survival (9). Initial observations of severe lung necrosis, hyperinflammation, vascular damage, thrombosis, and coagulation prompted therapeutic trials targeting classical platelet activation or the coagulation cascade (4, 10). Microvascular thrombosis of the pulmonary vasculature and other vascular beds is frequently observed in COVID‐19 (11). Patients in the intensive care unit with COVID-19 exhibit an elevated risk of ischemic stroke and disseminated intravascular coagulation, as indicated by increased C-reactive protein, D-dimer, and P-selectin levels (12). Notably, antiplatelet and anticoagulant trials did not consistently demonstrate a beneficial effect, and the lack of efficacy often coincided with the timing of infection and inflammation levels (13). The pathophysiology of COVID-19 has been explored in recent reviews (reviewed in (3, 14, 15). While evidence confirms inevitable platelet activation during COVID-19, the direct infectivity of platelets remains uncertain.

## Functions of platelets

Platelets, or thrombocytes, represent indispensable small blood cells crucial for hemostasis, aimed at preventing bleeding. Originating from megakaryocytes, platelets assume a pivotal role in the coagulation cascade (16). Their primary functions encompass the formation of blood clots to staunch wounds, thereby averting excessive bleeding. Platelets exhibit adherence to damaged blood vessels, releasing signaling chemicals that attract additional platelets, thereby instigating clot formation. Furthermore, platelets house granules containing clotting factors and growth factors imperative for tissue repair (17). Deviations in platelet function may precipitate bleeding disorders or hypercoagulability. Regular assessment of platelet levels through blood tests is standard practice. Disorders affecting platelets span thrombocytosis, characterized by elevated platelet counts, and thrombocytopenia, indicative of low platelet levels. In addition to their well-established hemostatic role, emerging research underscores the active involvement of platelets in immune responses.

Platelets promote hemostasis through a series of sequential processes: adhesion, activation, and aggregation (18). Following vascular injury in classical hemostasis, the injured vessel initiates vasoconstriction to limit blood loss. Subsequently, platelets adhere to the injured vessel wall, undergo activation, and form aggregates, constituting the primary and secondary platelet plug. This plug is further stabilized by a dense fibrin mesh formed through the coagulation cascade. Platelet activation is triggered by binding to both von Willebrand factor (vWF) and collagen (19). Upon activation, platelet granules are released, mediated by GPVI, leading to an increase in platelet activators such as ADP (adenosine diphosphate; from dense δ-granules), vWF (from α-granules, in addition to endothelial cell-derived vWF), and thromboxane A2 (20). These activators can, in turn, activate neighboring platelets. The binding of ADP and other potent platelet activators to their receptors induces platelet aggregation. Through an internal signaling mechanism, ADP binds to P2Y1 or P2Y12 receptors, resulting in a conformational change of the αIIbβ3 receptor on the platelet surface. In its active conformation, this receptor binds fibrinogen, facilitating platelet aggregation to form a thrombus and prevent bleeding (21).

## Platelets and COVID-19

In cases of COVID-19 pneumonia, researchers have observed elevated levels of platelet-granulocyte and platelet–monocyte aggregates (22). Based on previous autopsy studies, high platelet reactivity was linked with dispersed thrombosis in multiple organs, which suggested that SARS-CoV-2-mediated platelet activation, in turn, contributed to the pathophysiology of COVID-19 (23–26). Patients hospitalized with COVID‐19 have reported thrombocytopenia, and lower platelet counts have been associated with more adverse clinical outcomes (27). A meta-analysis comprising 31 studies and 7613 participants revealed a reduced platelet count in severe COVID‐19 cases, correlating with a 3-fold increase in the risk of developing severe COVID‐19 (28).

Increased plasma levels of proteins that could also be platelet-derived, such as sCD40LG (CD154), TxB2, P-selectin, and vWF, have confirmed that platelets are being activated during COVID-19. Consistently, platelet activation is evident by increased expression of P-selectin CD40 and CD63 on the surface of platelets from patients with COVID-19. A meta-analysis of 7,613 COVID-19 patients revealed that patients with severe disease had a lower platelet count than those with non-severe disease. Additionally, the non-survivors had a much lower platelet count than the survivors. However, not all studies have found platelet counts to be a predictor of COVID-19 mortality (29). Platelet activation was obvious in individuals with severe COVID-19, as measured by surface expression of P-selectin and CD63, and it was substantially linked with D-dimer levels. Soluble P-selectin levels were considerably higher in ICU patients than in non-ICU patients (30, 31). Furthermore, increased concentrations of both types of microvesicles (EV) (CD41+EV and annexin V+ CD41+ EV) per platelet in both patients with non-severe and severe COVID-19 compared with healthy controls were observed, suggesting the contribution of platelets derived MVs to COVID-19 disease pathology (31). Patients with COVID-19 exhibit elevated levels of platelet–neutrophil aggregates and increased neutrophil extracellular traps (NETs) (32, 33). These factors can contribute to the development of immunothrombosis, leading to thromboembolic complications. Ultimately, this process results in platelet consumption and thrombocytopenia, both of which are correlated with heightened mortality (27, 34). In contrast, there are studies supporting antiviral phenotype of platelets in various viral infections, including COVID-19, regulated through IFITs/IFITM3 (35, 36).

## Proposed platelet receptors interact with SARS-CoV-2

Platelets possess an array of receptors, including Cluster of differentiation(CD)40L, toll-like receptor, and the Fc receptor for IgG (FcyRIIa), enabling them to respond to stimuli (37). In other viral infections, such as Dengue haemorrhagic fever, antibody-coated virions induce substantial platelet activation through an FcγRIIa-dependent mechanism (38, 39). Similarly, Influenza H1N1 (40) and some Bunyaviruses like Crimea-Congo Haemorrhagic fever (41) induce platelet activation via FcγRIIa. However, the direct interaction mechanisms between SARS‐CoV‐2 and platelets or megakaryocytes remain contentious.

Shen et al., using RT-PCR assay, reported the detection of CD147, GRP78, KREMEN1, cathepsin L, NRP1, and ASGR1 in megakaryoblast cell line (MEG-01), and CD147, GRP78, KREMEN1, and ASGR1 in platelets. Notably, ACE2 was not detected in MEG-01 cell line or platelets (42, 43), suggesting SARS-CoV-2 may employ receptors other than ACE2 for interaction. Structural studies have proposed CD26 as another SARS-CoV-2 receptor, although its expression on platelets is debated (24, 43). Tang et al. demonstrated that the envelope (E) protein of SARS-CoV-2 enhances platelet activation and thrombosis through a CD36/p38 MAPK/NF-κB signaling axis (44). Additionally, platelets express a multitude of immune receptors, including CD40L, Toll-like receptors (TLRs), and the Fc receptor for IgG (FcγRIIA) (37). In line with this, a few other studies indicated that the E protein can physically interact with the TLR2 transmembrane receptor, stimulating NF-κB transcription and CXCL8 production (45, 46). Additionally, the viral Spike (S) protein independently binds with CD42b and stimulates platelets (47). Carnevale and colleagues provided evidence that SARS-CoV-2 uses the S protein to activate platelets via TLR4, leading to Nox2-related oxidative stress and a prothrombotic phenotype (48).

Another clinical study demonstrated that signaling through FcγRIIA and the C5a-C5aR pathway mediate platelet hyperactivation in COVID-19 (49). Puhm et al. demonstrated that TF from SARS-CoV-2–infected cells activates thrombin, signaling PARs on platelets, with potential implications for COVID-19 coagulation (50). Ito and colleagues proposed platelet αIIbβ3 integrin binds to the SARS-CoV-2 S protein of the alpha strain but not wild type and omicron strains (51).

**Figure 1:**
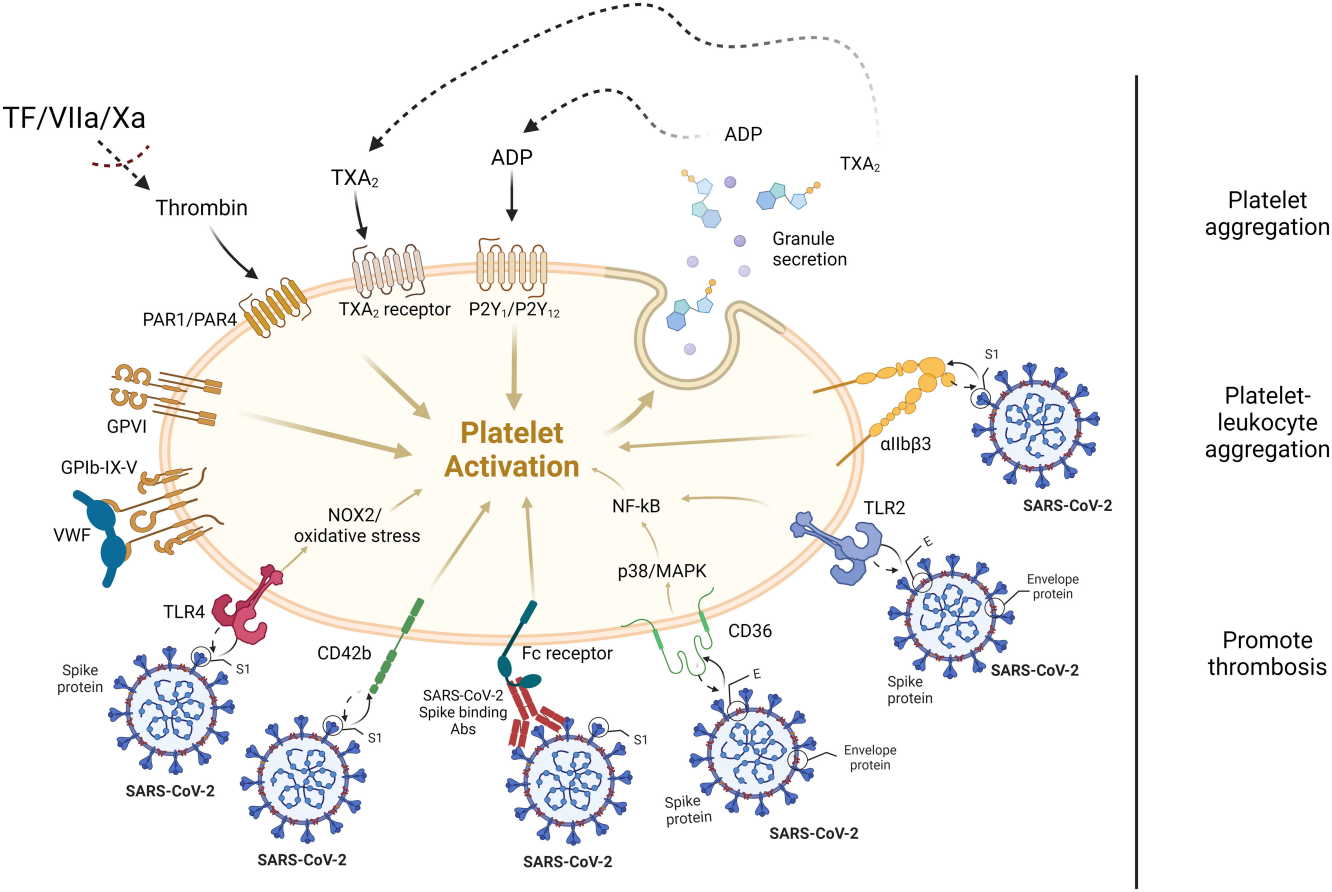
Proposed platelet receptors interaction with SARS-CoV-2. *In vitro* studies proposed that SARS-CoV-2 virus S and E proteins interact with platelet surface receptors, such as CD42b, TLR4, Fc receptor, CD36, TLR2, and αIIbβ3 integrin. Most soluble agonists released by activated platelets, such as adenosine diphosphate (ADP), thromboxane A2 (TxA2) as well as TF/FVIIa/Xa-mediated thrombin trigger platelet activation through GPCRs. For instance, elevated levels of thrombin in the circulatory system due to the activation of coagulation cascade, also activates the platelets through PAR1/PAR4 receptors. S, spike protein; E, envelope protein.

Despite the distinct biological characteristics of SARS-CoV-2 Omicron, it leads to platelet activation and desensitization, similar to observations with the Delta variant. Omicron is also found in platelets from severe patients, inducing selective autophagy. However, the mechanisms of intraplatelet processing of Omicron cargo differ from Delta, suggesting that S protein mutations modify virus-platelet interactions (52). Although several receptors on platelet surface (**Figure 1**), besides ACE2, proposed for viral entry in platelets, due to the limited *in vivo* experimental evidence, further studies are required to substantiate these claims.

## Controversies in COVID-19 and direct infection of platelets

Initial investigations proposed the direct invasion of platelets by SARS-CoV-2, impacting their functionality (53). Autopsies reported the presence of SARS-CoV-2 viral particles in both megakaryocytes and platelets (54). Notably, Zhang et al. reported ACE2 expression on the platelet surface (55), but this observation was not consistently replicated in other studies (24, 31, 54, 56). The conflicting findings raise questions about the interaction between SARS-CoV-2 and platelets through ACE2. RNA-seq and western blot analyses revealed no evidence of ACE2 or TMPRSS2 in CD45‐depleted platelets from both COVID‐19 patients and healthy individuals (24). Zaid et al. conducted a similar study and also reported the absence of ACE2 on platelets from COVID‐19 patients and healthy volunteers (31). A retrospective survey of plasma samples from severe and non-severe COVID‐19 patients revealed increased thrombosis and elevated levels of sP‐selectin, sGPVI, RANTES, and PF4 during platelet activation (43). In contrast, Zhang et al. reported significant expression of ACE2 and TMPRSS2 mRNA and protein on platelets from healthy individuals and mice. Furthermore, *in vitro* and *in vivo* experiments using humanized ACE2 transgenic mice demonstrated that SARS‐CoV‐2 and its S protein directly activate platelets (55). Another study by Koupenova et al. revealed that SARS‐CoV‐2 induces programmed cell death in platelets, leading to internalization of virions attached to microparticles, bypassing ACE2 (57). This internalization results in rapid digestion, apoptosis, necroptosis, and extracellular vesicle release, contributing to dysregulated immunity and thrombosis. However, these conflicting observations raise questions about the direct interaction between SARS‐CoV‐2 and platelets and the concept of direct platelet infectivity.

## Conclusion

The current body of evidence underscores the ambiguity surrounding the direct interaction between SARS-CoV-2 and platelets. The disparate observations of viral load within platelets from COVID-19 patients, coupled with the identification of numerous alternative receptors on the platelet surface, have contributed to the uncertainty. *In vitro* and *in vivo* experimental studies as well as proteomic and transcriptomic analyses of platelets from COVID-19 patients and mice infected with SARS-CoV-2 further highlight the unlikelihood of direct platelet infectivity for the activation of platelets by SARS-CoV-2 viral proteins. Amidst this uncertainty, critical or fatal COVID-19 cases have exhibited various pathological abnormalities, including endothelial dysfunction, cytokine storms, and the formation of NETs. These abnormalities may be exacerbated by platelet hyperactivity, resulting from the aggregation of platelets to activated endothelial cells and neutrophils.

## Author contributions

SS: Conceptualization, Data curation, Methodology, Resources, Supervision, Validation, Visualization, Writing – original draft, Writing – review & editing. NM: Visualization, Writing – review & editing. AJ: Visualization, Writing – review & editing.
